# The genome sequence of the small elephant hawk moth, *Deilephila porcellus *(Linnaeus, 1758)

**DOI:** 10.12688/wellcomeopenres.17740.1

**Published:** 2022-03-08

**Authors:** Douglas Boyes, University of Oxford and Wytham Woods Genome Acquisition Lab, Darwin Tree of Life Barcoding collective, Wellcome Sanger Institute Tree of Life programme, Wellcome Sanger Institute Scientific Operations: DNA Pipelines collective, Tree of Life Core Informatics collective, Laura Sivess, Darwin Tree of Life Consortium

**Affiliations:** 1 UK Centre for Ecology & Hydrology, Wallingford, UK; 2 Department of Life Sciences, Natural History Museum, London, UK

**Keywords:** Deilephila porcellus, small elephant hawk moth, genome sequence, chromosomal, Lepidoptera

## Abstract

We present a genome assembly from an individual male *Deilephila porcellus *(the small elephant hawk moth; Arthropoda; Insecta; Lepidoptera; Sphingidae). The genome sequence is 402 megabases in span. The majority of the assembly (99.99%) is scaffolded into 29 chromosomal pseudomolecules, with the Z sex chromosome assembled.

## Species taxonomy

Eukaryota; Metazoa; Ecdysozoa; Arthropoda; Hexapoda; Insecta; Pterygota; Neoptera; Endopterygota; Lepidoptera; Glossata; Ditrysia; Bombycoidea; Sphingidae; Macroglossinae; Macroglossini; Deilephila; *Deilephila porcellus* (Linnaues, 1758) (NCBI:txid644661).

## Background

*Deilephila porcellus* (small elephant hawk-moth) is characterised by striking pink and sand markings and is distributed across Europe, reaching as far East as China. Often confused with *Deilephila elpenor* (elephant hawk-moth), *Deilephila porcellus* can be identified most easily by a slightly smaller wingspan (40–45mm), brighter colouration and lack of the longitudinal pink abdominal stripe, typical of *D. elpenor*.

*Deilephila porcellus* is widespread throughout Britain, of rather local distribution in Southern England and Wales and scarce in Scotland and Northern England. This species flies from May to July and can be found in a range of open habitats including grassland, heathland, sand dunes and shingle beaches ( Waring *et al.*, 2017). Adults are generalists, nocturnally feeding on the nectar of numerous flowering plants, including Rhododendron and Honeysuckle. Orchids are frequently visited for nectar; the relative frequency of different hawk-moth pollinators, with their differing proboscis lengths, has been shown to select for different spur lengths in the lesser butterfly orchid ( *Platanthera bifolia*). in open areas in Sweden, the relatively short-tongued *Deilephila porcellus* is the most frequent pollinator and the orchid’s spurs are correspondingly short when compared to woodland populations, mainly pollinated by the long-tongued *Sphinx ligustri ( Boberg *et al.*, 2014)*. Caterpillars, which primarily feed on bedstraws ( *Galium*), emerge from June to September, and vary in colouration from brown to grey-green with large eyespots situated towards the anterior end. Functionally, eyespots and behaviour act to deter avian predation; when threatened, larvae widen anterior segments of the body, adopting defensive postures thought to mimic snakes, thus reducing incidence of attacks ( Hossie & Sherratt, 2013; Poulton, 1890; ). The full lifecycle takes one year to complete, with pupae over-wintering in cocoons beneath larval food plants or just below the surface of the leaf litter.

Here we present a genome sequence for *D. porcellus*, generated as part of the Darwin Tree of Life Project.

## Genome sequence report

**Figure 1.**
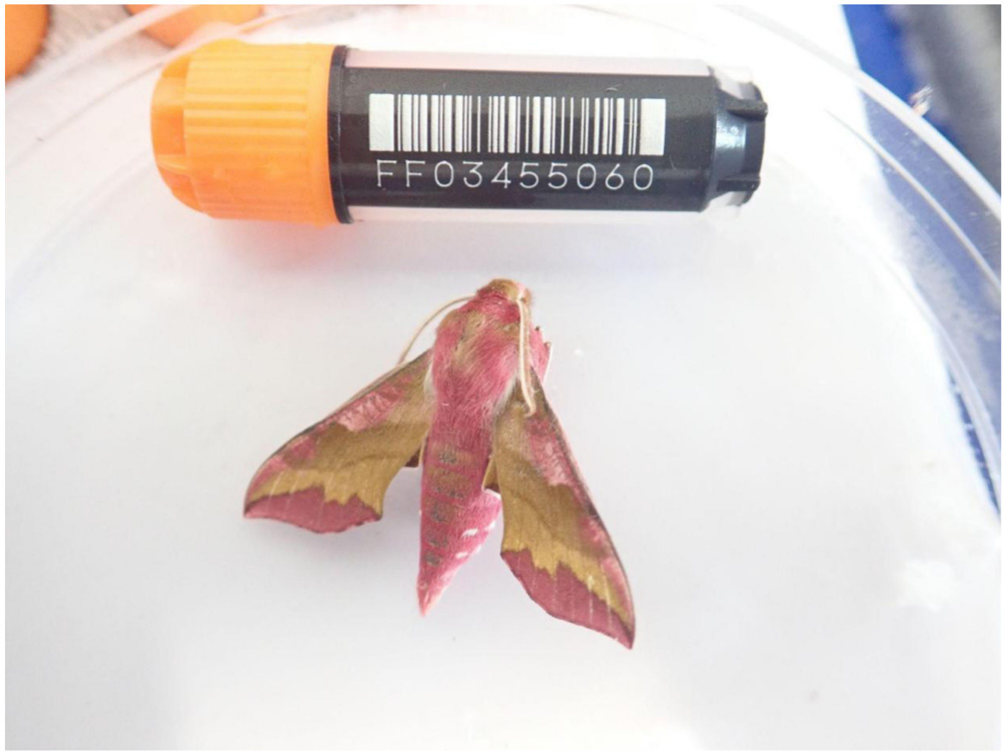
Image of the *Deilephila porcellus* specimen taken prior to preservation and processing.

The genome was sequenced from a single male *D. porcellus* ( Figure 1) collected from Wytham Woods, Oxfordshire, UK (latitude 51.772, longitude -1.337). A total of 40-fold coverage in Pacific Biosciences single-molecule HiFi long reads and 92-fold coverage in 10X Genomics read clouds were generated. Primary assembly contigs were scaffolded with chromosome conformation Hi-C data. Manual assembly curation corrected 4 missing/misjoins and removed 1 haplotypic duplications, reducing the scaffold number by 9.09%.

**Figure 2.**
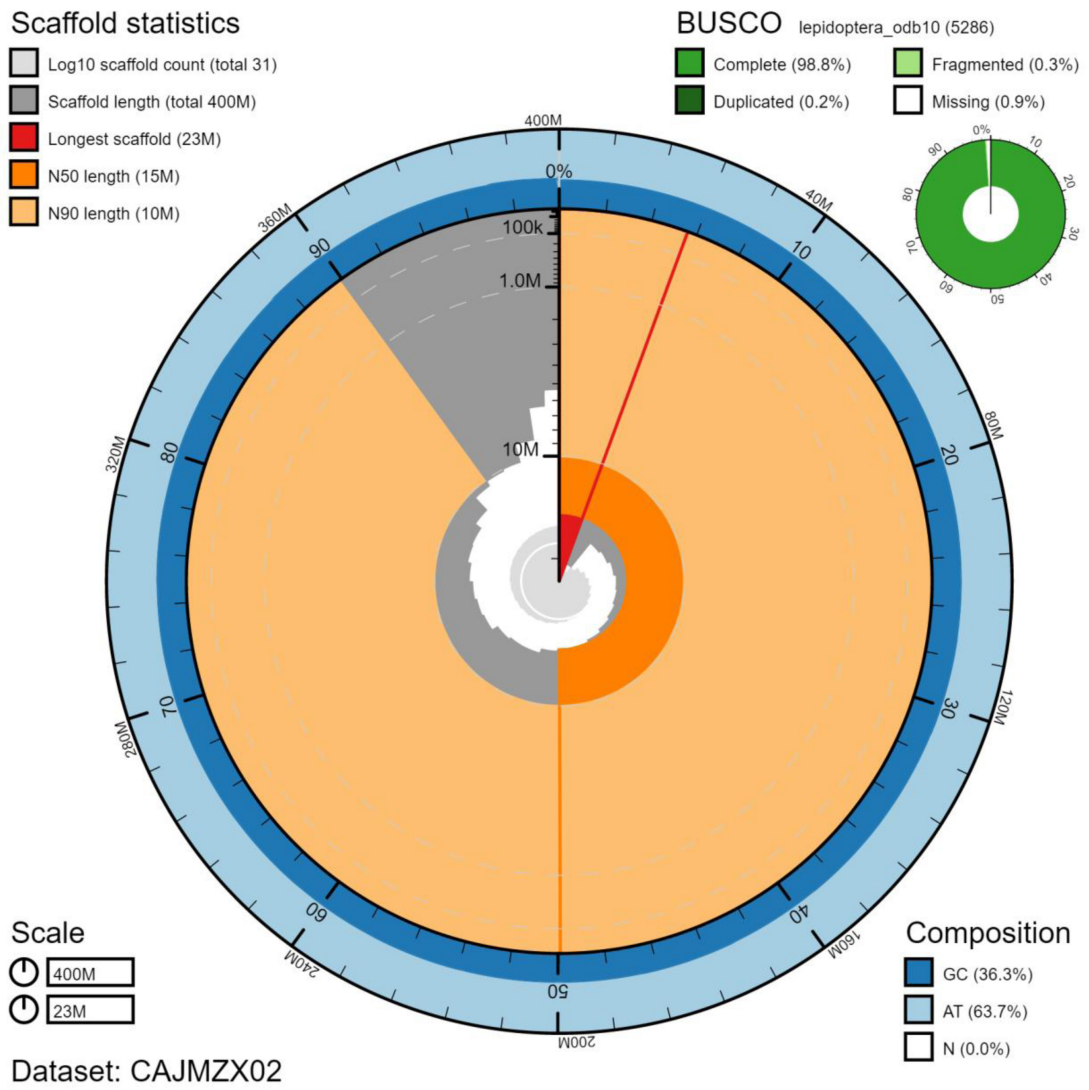
Genome assembly of *Deilephila porcellus*, ilDeiPorc1.2: metrics. The main plot is divided into 1,000 size-ordered bins around the circumference with each bin representing 0.1% of the 402,071,895 bp assembly. The distribution of chromosome lengths is shown in dark grey with the plot radius scaled to the longest chromosome present in the assembly (22,662,151 bp, shown in red). Orange and pale-orange arcs show the N50 and N90 chromosome lengths (15,067,504 and 10,104,753 bp), respectively. The pale grey spiral shows the cumulative chromosome count on a log scale with white scale lines showing successive orders of magnitude. The blue and pale-blue area around the outside of the plot shows the distribution of GC, AT and N percentages in the same bins as the inner plot. A summary of complete, fragmented, duplicated and missing BUSCO genes in the lepidoptera_odb10 set is shown in the top right. An interactive version of this figure is available at https://blobtoolkit.genomehubs.org/view/ilDeiPorc1.2/dataset/CAJMZX02/snail.

The final assembly has a total length of 402 Mb in 30 sequence scaffolds with a scaffold N50 of 15.1 Mb ( Table 1). Of the assembly sequence, 99.99% was assigned to 29 chromosomal-level scaffolds, representing 28 autosomes (numbered by sequence length), and the Z sex chromosome ( Figure 2– Figure 5; Table 2). The assembly has a BUSCO ( Simão *et al.*, 2015) completeness of 98.8% (single, 98.5%, duplicated 0.2%) using the lepidoptera_odb10 reference set (n=5286). While not fully phased, the assembly deposited is of one haplotype. Contigs corresponding to the second haplotype have also been deposited.

**Table 1.** Genome data for *Deilephila porcellus*, ilDeiPorc1.2.

*Project accession data*	
Assembly identifier	ilDeiPorc1.2
Species	*Deilephila porcellus*
Specimen	ilDeiPorc1
NCBI taxonomy ID	644661
BioProject	PRJEB42950
BioSample ID	SAMEA7520522
Isolate information	Male, head/thorax (Hi-C), abdomen (genome assembly)
*Raw data accessions*	
PacificBiosciences SEQUEL II	ERR6406201, ERR6412027
10X Genomics Illumina	ERR6054401-ERR6054404
Hi-C Illumina	ERR6054400
*Genome assembly*	
Assembly accession	GCA_905220455.2
*Accession of alternate haplotype*	GCA_905220465.1
Span (Mb)	402
Number of contigs	35
Contig N50 length (Mb)	14.9
Number of scaffolds	30
Scaffold N50 length (Mb)	15.1
Longest scaffold (Mb)	20.4
BUSCO * genome score	C:98.8%[S:98.5%,D:0.2%],F:0.3%, M:0.9%,n:5286

*BUSCO scores based on the lepidoptera_odb10 BUSCO set using v5.1.2. C= complete [S= single copy, D=duplicated], F=fragmented, M=missing, n=number of orthologues in comparison. A full set of BUSCO scores is available at https://blobtoolkit.genomehubs.org/view/ilDeiPorc1.2/dataset/CAJMZX02/busco.

**Figure 3.**
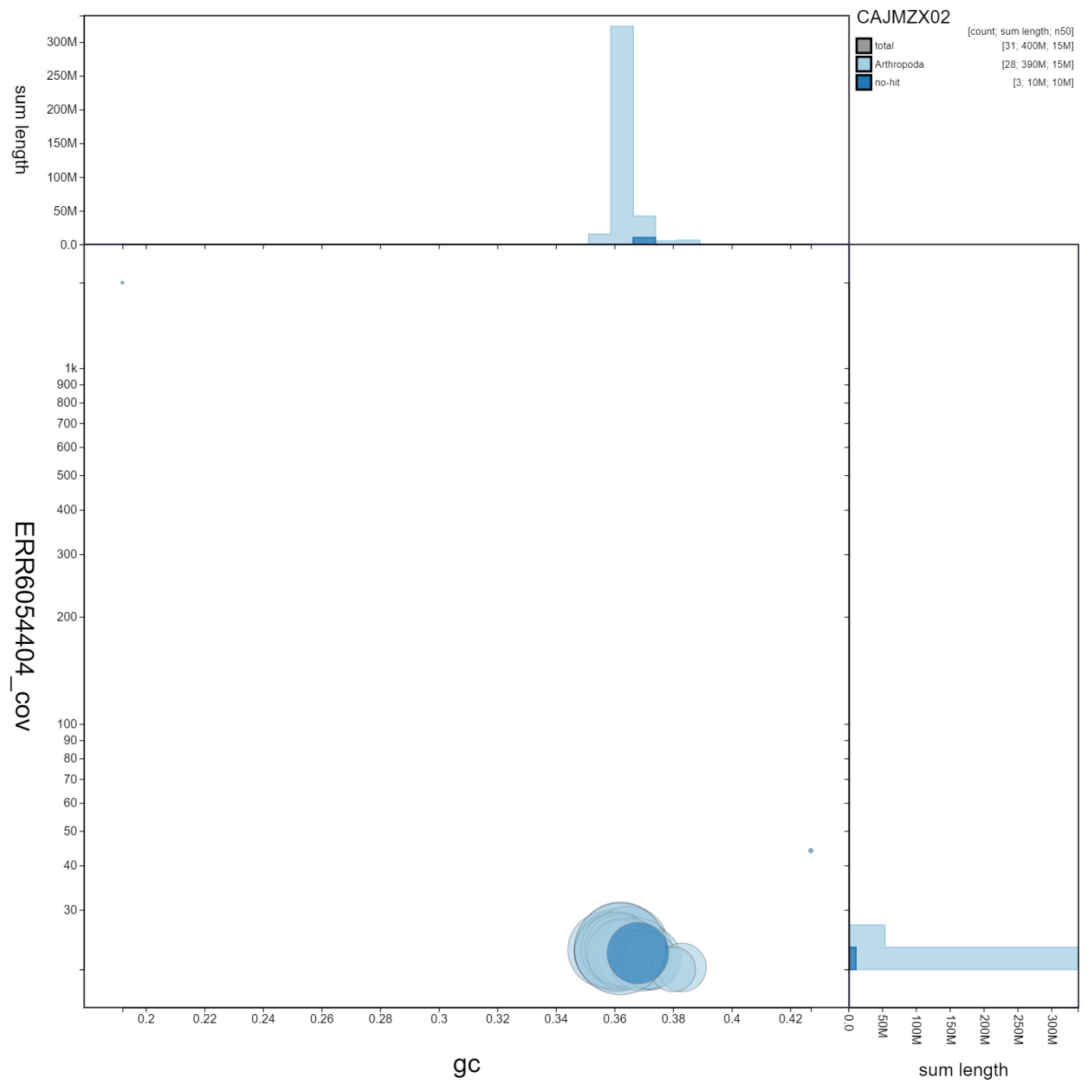
Genome assembly of *Deilephila porcellus*, ilDeiPorc1.2: GC coverage. BlobToolKit GC-coverage plot. Scaffolds are coloured by phylum. Circles are sized in proportion to scaffold length. Histograms show the distribution of scaffold length sum along each axis. An interactive version of this figure is available at https://blobtoolkit.genomehubs.org/view/ilDeiPorc1.2/dataset/CAJMZX02/blob.

**Figure 4.**
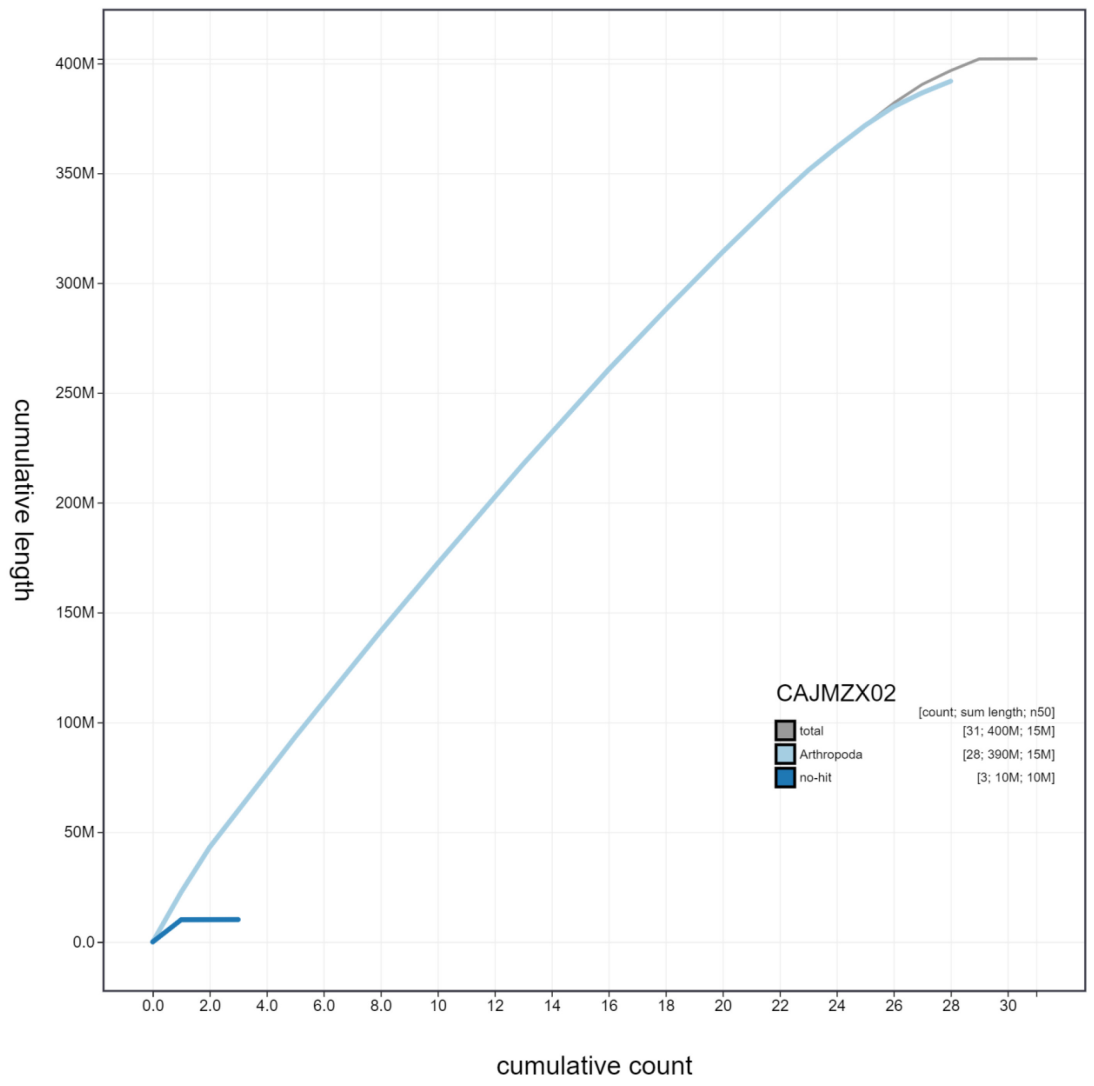
Genome assembly of *Deilephila porcellus*, ilDeiPorc1.2: cumulative sequence. BlobToolKit cumulative sequence plot. The grey line shows cumulative length for all scaffolds. Coloured lines show cumulative lengths of scaffolds assigned to each phylum using the buscogenes taxrule. An interactive version of this figure is available at https://blobtoolkit.genomehubs.org/view/ilDeiPorc1.2/dataset/CAJMZX02/cumulative.

**Figure 5.**
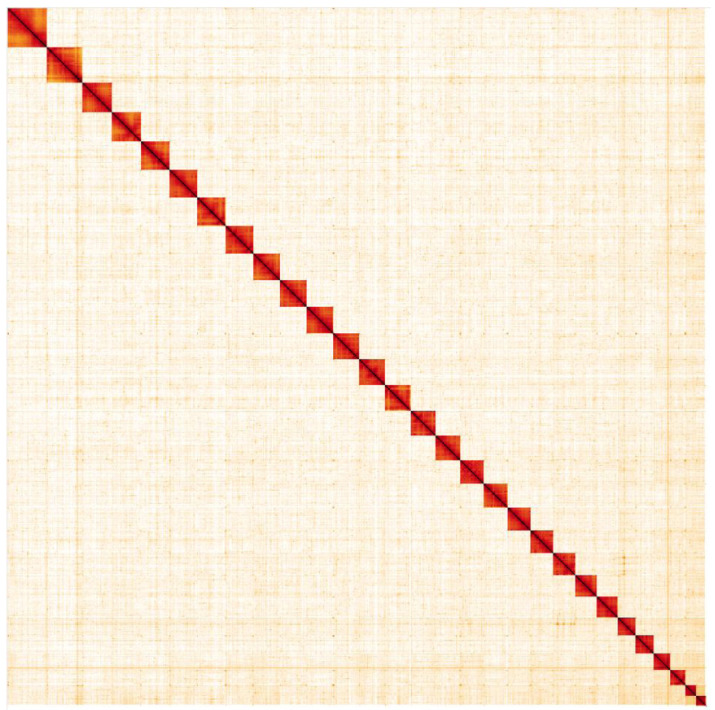
Genome assembly of *Deilephila porcellus*, ilDeiPorc1.2: Hi-C contact map. Hi-C contact map of the ilDeiPorc1.2 assembly, visualised in HiGlass. Chromosomes are given in size order from left to right and top to bottom.

**Table 2.** Chromosomal pseudomolecules in the genome assembly of *Deilephila porcellus* ilDeiPorc1.2.

INSDC accession	Chromosome	Size (Mb)	GC%
LR999971.1	1	20.41	36.2
LR999972.1	2	17.03	36.1
LR999973.1	3	16.78	36.4
LR999974.1	4	16.43	36.4
LR999975.1	5	16.18	36.2
LR999976.1	6	16.07	36.5
LR999977.1	7	15.87	36.0
LR999978.1	8	15.64	36.1
LR999979.1	9	15.35	35.7
LR999980.1	10	15.14	36.2
LR999981.1	11	15.07	35.9
LR999982.1	12	14.89	35.9
LR999983.1	13	14.45	36.1
LR999984.1	14	14.44	35.9
LR999985.1	15	14.25	36.1
LR999986.1	16	13.66	36.3
LR999987.1	17	13.52	36.2
LR999988.1	18	13.31	36.4
LR999989.1	19	12.99	36.8
LR999990.1	20	12.67	36.6
LR999991.1	21	12.58	36.5
LR999992.1	22	11.95	36.2
LR999993.1	23	10.52	37.2
LR999994.1	24	10.10	36.8
LR999995.1	25	9.95	36.9
LR999996.1	26	8.52	37.2
LR999997.1	27	6.29	38.3
LR999998.1	28	5.31	38.0
LR999970.1	Z	22.66	36.2
LR999999.2	MT	0.02	19.3
-	Unplaced	0.04	42.7

## Methods

### Sample acquisition and nucleic acid extraction

A male *D. porcellus* (ilDeiPorc1) was collected from Wytham Woods, Oxfordshire, UK (latitude 51.772, longitude -1.337) by Douglas Boyes, University of Oxford, using a light trap. The specimens were identified by the same individual and snap-frozen on dry ice.

DNA was extracted at the Tree of Life laboratory, Wellcome Sanger Institute. The ilDeiPorc1 sample was weighed and dissected on dry ice with tissue set aside for Hi-C sequencing. Abdomen tissue was cryogenically disrupted to a fine powder using a Covaris cryoPREP Automated Dry Pulveriser, receiving multiple impacts. Fragment size analysis of 0.01–0.5 ng of DNA was then performed using an Agilent FemtoPulse. High molecular weight (HMW) DNA was extracted using the Qiagen MagAttract HMW DNA extraction kit. Low molecular weight DNA was removed from a 200-ng aliquot of extracted DNA using 0.8X AMpure XP purification kit prior to 10X Chromium sequencing; a minimum of 50 ng DNA was submitted for 10X sequencing. HMW DNA was sheared into an average fragment size between 12–20 kb in a Megaruptor 3 system with speed setting 30. Sheared DNA was purified by solid-phase reversible immobilisation using AMPure PB beads with a 1.8X ratio of beads to sample to remove the shorter fragments and concentrate the DNA sample. The concentration of the sheared and purified DNA was assessed using a Nanodrop spectrophotometer and Qubit Fluorometer and Qubit dsDNA High Sensitivity Assay kit. Fragment size distribution was evaluated by running the sample on the FemtoPulse system.

### Sequencing

Pacific Biosciences HiFi circular consensus and 10X Genomics Chromium read cloud sequencing libraries were constructed according to the manufacturers’ instructions. Sequencing was performed by the Scientific Operations core at the Wellcome Sanger Institute on Pacific Biosciences SEQUEL II (HiFi) and Illumina HiSeq X (10X) instruments. Hi-C data were generated from head/thorax tissue of ilDeiPorc1 using the Arima v2 kit and sequenced on HiSeq X.

### Genome assembly

Assembly was carried out with Hifiasm ( Cheng *et al.*, 2021). Haplotypic duplication was identified and removed with purge_dups ( Guan *et al.*, 2020). One round of polishing was performed by aligning 10X Genomics read data to the assembly with longranger align, calling variants with freebayes ( Garrison & Marth, 2012). The assembly was then scaffolded with Hi-C data ( Rao *et al.*, 2014) using SALSA2 ( Ghurye *et al.*, 2019). The assembly was checked for contamination and corrected using the gEVAL system ( Chow *et al.*, 2016) as described previously ( Howe *et al.*, 2021). Manual curation was performed using gEVAL, HiGlass ( Kerpedjiev *et al.*, 2018) and Pretext. The mitochondrial genome was assembled using MitoHiFi ( Uliano-Silva *et al.*, 2021), which performed annotation using MitoFinder ( Allio *et al.*, 2020). The genome was analysed and BUSCO scores generated within the BlobToolKit environment ( Challis *et al.*, 2020). Table 3 contains a list of all software tool versions used, where appropriate.

**Table 3.** Software tools used.

Software tool	Version	Source
Hifiasm	0.12	Cheng *et al.*, 2021
purge_dups	1.2.3	Guan *et al.*, 2020
SALSA2	2.2	Ghurye *et al.*, 2019
longranger align	2.2.2	https://support.10xgenomics.com/genome-exome/ software/pipelines/latest/advanced/other-pipelines
freebayes	1.3.1-17-gaa2ace8	Garrison & Marth, 2012
MitoHiFi	1.0	Uliano-Silva *et al.*, 2021
gEVAL	N/A	Chow *et al.*, 2016
PretextView	0.1.x	https://github.com/wtsi-hpag/PretextView
HiGlass	1.11.6	Kerpedjiev *et al.*, 2018
BlobToolKit	2.6.4	Challis *et al.*, 2020

## Data availability

European Nucleotide Archive: Deilephila porcellus (small elephant hawk-moth). Accession number PRJEB42950; https://identifiers.org/ena.embl/PRJEB42950.

The genome sequence is released openly for reuse. The *D. porcellus* genome sequencing initiative is part of the Darwin Tree of Life (DToL) project. All raw sequence data and the assembly have been deposited in INSDC databases. The genome will be annotated and presented through the Ensembl pipeline at the European Bioinformatics Institute. Raw data and assembly accession identifiers are reported in Table 1.
