# Supplementary material for: The genome sequence of the small elephant hawk moth, Deilephila porcellus (Linnaeus, 1758)
Source: Wellcome Open Res. 2022 Mar 8;7:80. [Version 1] doi: 10.12688/wellcomeopenres.17740.1 (PMC9021678; doi:10.12688/wellcomeopenres.17740.1)
Supplement: Peer review history

# Peer review: Reviewer response for version 1

**DOI:** 10.21956/wellcomeopenres.19631.r49246

**Published:** 2022-04-11

**Authors:** Surya Saha

**Affiliations:** 1 Boyce Thompson Institute for Plant Research, Ithaca, NY, USA

The rapid release of data for use by the community is commendable and the Wellcome Open Research Data Notes are an excellent practice adopted by the DToL project. This manuscript is well-written and presents a high quality and contiguous arthropod genome assembly with DNA sourced from a single individual which is the gold standard for genome assembly. The NCBI Bioproject is well structured and provides access to both the primary and alternate haplotype assemblies.

 I only have minor comments:

1. Although the versions and tools are mentioned, the methods lack enough details for reasonable reproducibility. The review from Martin already describes these lacunae in sufficient detail but I will reiterate them here for the sake of completeness. Parameters used for execution (even if they were the defaults) need to be reported for readers. Sharing a script with commands to reproduce the major steps in the assembly using a github repository release with a zenodo DOI might be a solution worth considering.
2. Figures 3 and 4 don’t provide a lot of insights for genomes of this quality.
3. Using a kmer mining toolkit like KAT ( https://github.com/TGAC/KAT) can help uncover the heterozygosity and repetitiveness in the assembly considering this is not a completely phased assembly. This information might have already been generated with Meryl ( https://github.com/VGP/vgp-assembly/tree/master/pipeline/meryl).
4. Was RepeatMasker run on the assembly as a part of the VGP pipeline ( https://github.com/VGP/vgp-assembly/tree/master/pipeline). A summary table of repeat elements can be helpful for understanding the architecture of this genome.

Are sufficient details of methods and materials provided to allow replication by others?

Partly

Is the rationale for creating the dataset(s) clearly described?

Yes

Are the datasets clearly presented in a useable and accessible format?

Yes

Are the protocols appropriate and is the work technically sound?

Yes

Reviewer Expertise:

genome assembly and annotation, i5k, Ag100Pest, AgriVectors, Hemipteran genomics

I confirm that I have read this submission and believe that I have an appropriate level of expertise to confirm that it is of an acceptable scientific standard.

# Peer review: Reviewer response for version 1

**DOI:** 10.21956/wellcomeopenres.19631.r49245

**Published:** 2022-03-22

**Authors:** Martin Pippel

**Affiliations:** 1 Max Planck Institute of Molecular Cell Biology and Genetics, Dresden, Germany

“The genome sequence of the small elephant hawk moth, *Deilephila porcellus*” from Boyes *et al. *describes a high-quality genome assembly based on PacBio HiFi reads, 10X Genomics read clouds and HiC data.  

 The data note is well structured and based on the described methods and the publicly available sequencing data also reproducible by the scientific community. Already the contig assembly shows a very high contiguity, indeed 25 out 29 chromosomal-level scaffolds are assembled into single contigs.

**Methods:**

 Genome assembly methods are a bit short and it could be hard to reproduce the assembly without further documentation of the used program arguments. Even if all tools were run in default mode, it would be worth mentioning. Some of the used tools, such as purge_dups depend itself on other programs like minimap2. But those could not be found in Table 3. I could not find any information if all variants from Freebayes were used for the error polishing or if a variant filtering step was applied beforehand.

 I do only have some minor comments and suggestions:

- I could not find the HiC read coverage in the Data Note.
- The plots from Figures 3 and 4 are for such a high-contiguous assembly not very informative.
- Additional statistics about the sequencing data (e.g. read length N50 of HiFi reads and 10X read clouds, kmer based genome size and heterozygosity estimates) and the final assembly (e.g. merqury QV scores, repeat content) would be nice to see as well.
- Error-polishing strategies of HiFi-based assemblies are currently under debate. I assume you applied bcftools consensus on the filtered Freebayes VCF files similar to the VGP assembly pipeline ( https://github.com/VGP/vgp-assembly/tree/master/pipeline). How big was the improvement based in the QV-score? Were the alternate contigs included in the longranger alignment step?
- More recent versions of hifiasm offer a greatly improved phasing module based on HiC reads. Due to the high HiFi coverage of 40X it would be interesting to see if this could create fully phased chromosomes.
- I was surprised that only 5 scaffolds contain the telomer motifs at both ends, 18 scaffolds (plus 1 contig) show one telomer sequence, and 6 scaffolds do not show the telomer sequence. Could it be possible that purge_dups trimmed off those sequence motifs from the end of the contigs or even removed those contigs?

Are sufficient details of methods and materials provided to allow replication by others?

Partly

Is the rationale for creating the dataset(s) clearly described?

Yes

Are the datasets clearly presented in a useable and accessible format?

Yes

Are the protocols appropriate and is the work technically sound?

Yes

Reviewer Expertise:

genome assembly, VGP, Bat1K, ERGA, eurofish, bioinformatics

I confirm that I have read this submission and believe that I have an appropriate level of expertise to confirm that it is of an acceptable scientific standard.
